# The built environment as determinant of childhood obesity: A systematic literature review

**DOI:** 10.1111/obr.13385

**Published:** 2021-12-03

**Authors:** Diego Malacarne, Evangelos Handakas, Oliver Robinson, Elisa Pineda, Marc Saez, Leda Chatzi, Daniela Fecht

**Affiliations:** ^1^ MRC Centre for Environment and Health, School of Public Health Imperial College London London UK; ^2^ Centre for Health Economics & Policy Innovation (CHEPI), Imperial College Business School, and School of Public Health Imperial College London London UK; ^3^ Research Group on Statistics, Econometrics and Health (GRECS) University of Girona Girona Spain; ^4^ CIBER of Epidemiology and Public Health (CIBERESP) Madrid Spain; ^5^ Keck School of Medicine University of Southern California Los Angeles CA USA

**Keywords:** air pollution, children, STOP project, walkability

## Abstract

We evaluated the epidemiological evidence on the built environment and its link to childhood obesity, focusing on environmental factors such as traffic noise and air pollution, as well as physical factors potentially driving obesity‐related behaviors, such as neighborhood walkability and availability and accessibility of parks and playgrounds. Eligible studies were (i) conducted on human children below the age of 18 years, (ii) focused on body size measurements in childhood, (iii) examined at least one built environment characteristic, (iv) reported effect sizes and associated confidence intervals, and (v) were published in English language. A *z* test, as alternative to the meta‐analysis, was used to quantify associations due to heterogeneity in exposure and outcome definition. We found strong evidence for an association of traffic‐related air pollution (nitrogen dioxide and nitrogen oxides exposure, *p* < 0.001) and built environment characteristics supportive of walking (street intersection density, *p* < 0.01 and access to parks, *p* < 0.001) with childhood obesity. We identified a lack of studies that account for interactions between different built environment exposures or verify the role and mechanism of important effect modifiers such as age.

**Abbreviations**
BMI z‐score: BMI standardized for age and sex
BMI: body mass index
CDC: Centers for Disease Control and Prevention
ESCAPE: European Study of Cohorts for Air Pollution Effects
IOTF: International Obesity Task Force
NDVI: Normalized Difference Vegetation Index
NO_2_
: nitrogen dioxide
NO_x_
: nitrogen oxides
PIAMA: prevention and incidence of asthma and mite allergy birth cohort
PM_10_
: particulate matter with diameter less than 10 μm
PM_2.5_
: particulate matter with diameter less than 2.5 μm
PRISMA: Preferred Reporting Items for systematic Reviews and Meta‐Analysis
PROSPERO: International Prospective Register of Systematic Reviews
SO_2_
: sulfur dioxide
STAMINA: Standard Model Instrumentation for Noise Assessments
UK: United Kingdom
WHO: World Health Organization

## 1 INTRODUCTION

The prevalence of childhood obesity has more than tripled over the last four decades. Latest figures suggest that up to 30% of children in Europe are with overweight or obesity.^1^ The growing rate of children with overweight and obesity is the most important preventable public health crisis of the 21st century, with serious health, social, and economic implications. Obesity in childhood often persists into adulthood with severe consequences for health. An expanding set of chronic diseases has been linked to childhood obesity including increased risk of developing cardiovascular disease, type 2 diabetes, and certain cancers, as well as diminished mental health.^2^, ^3^, ^4^, ^5^

Obesity is preventable and reversible. Restricting energy intake and increasing energy expenditure have previously been the focus of prevention and treatment strategies. Most efforts and initiatives have, however, so far been unsuccessful at a population level, and a broadened approach is warranted.^6^ The causes of obesity are multifactorial ranging from individual, household, to policy settings. In this context, place‐based obesogenic factors are increasingly being recognized as important determinants of obesity, including the social context, the environment individuals live in, and behaviors linked to modern, urban living.^7^ In order to target place‐based mitigation approaches, interventions, and policy implementations, a clear understanding of the spatial context in which obesity determinants act is needed.^8^

The place we live in has increasingly been recognized as a strong determinant of health, including obesity.^9^ In this context, the term “built environment” has been coined to describe the physical and built infrastructure in which people live, learn, work, play, socialize, and travel.^10^ Within urban settings, the natural infrastructure is an integral part of the wider concept of the built environment. The built environment has strong influences on residents' behaviors, with physical activity and sedentary lifestyles being the most widely studied.^11^ Additionally, environmental pollution linked to the built environment such as air pollution and traffic also has strong impacts on urban health.^12^

This systematic review synthesizes the empirical evidence on the built environment as determinant of childhood obesity. We focused on environmental factors including traffic noise and air pollution, as well as physical factors potentially driving obesity‐related behaviors, including neighborhood walkability, and availability and accessibility of parks and playgrounds. Supported by a rigorous quality assessment and a focus on objectively measured built environment characteristics, we provide a quantitative synthesis of the updated evidence base with an emphasis on conceptual and methodological aspects and public health implications.

## 2 METHODS

### 2.1 Search strategy

We followed the Preferred Reporting Items for Systematic reviews and Meta‐Analysis (PRISMA) guidelines and registered the protocol with the International Prospective Register of Systematic Reviews (PROSPERO) database (registration number CRD42020170337). We used a comprehensive and reproducible search strategy to identify peer‐reviewed journal articles in the English language, published from inception until February 2020, focusing on three databases: EMBASE, MEDLINE, and Web of Science. A preliminary search identified relevant keywords and MeSH terms at the intersection of two concept clusters: “childhood obesity” and “built environment” (Table S1).

### 2.2 Eligibility criteria

Studies were eligible for inclusion if they met the following criteria: (1) Population: Children and/or adolescents under the age of 18 years; (2) Exposure: Objectively measured environmental and physical features of the built environment potentially linked to the onset of obesity; (3) Outcomes: Objectively measured and self‐reported body mass index (BMI) or BMI standardized for age and sex (BMI *z* score); (4) Study design: Observational studies (cross‐sectional and longitudinal) quantitatively assessing associations of outcome and exposure. We excluded studies that assessed the built environment as confounder only, those that used self‐reported perceived features of the built environment, and studies using controlled experiments in manipulated settings (Table S2). We also excluded studies with an explicit focus on the food environment as this was outside the scope of the review. After the removal of duplicates, articles were screened independently by two reviewers (D.M. and D.F.) against the eligibility criteria, using the online tool Covidence.^13^

### 2.3 Data extraction

Data extraction was performed independently by two reviewers (D.M. and E.H.), and discrepancies were mediated by D.F. Information was extracted on study characteristics (first author, year, study design, study area, sample size), participant characteristics (age, sex), exposure (built environment characteristic, data collection method), outcome measures (outcome, data collection methods, measure of association), individual‐ and area‐level confounders, and main findings (direction and magnitude of association, statistical significance).

### 2.4 Quality assessment

The quality of the eligible studies was assessed independently by two reviewers (D.M. and E.H.), and discrepancies were mediated by D.F. We used a modified Newcastle–Ottawa scale for quality assessment,^14^ which we adapted for the assessment of observational studies. The elements used for the assessment include (1) representativeness of the exposed population, (2) selection of the nonexposed population, (3) objective ascertainment of the exposure, (4) sample size, (5) appropriateness of considered confounding factors, (6) assessment of the outcome, and (7) statistical test used for analysis (Table S3). Stars were assigned for each criterion with a maximum of 12 stars. A score of 0–4 was defined as poor quality, 5–8 as fair quality, and 9–12 as good quality. Publication bias was assessed using a funnel plot.

### 2.5 Data synthesis

Due to the heterogeneity in exposure metrics and methodologies used across eligible studies, a meta‐analysis was not possible. Instead, we used an alternative methodology to assess and synthesize the strength of associations, the weighted *z* test.^15^ This approach has previously been used for systematic reviews on the built environment and health^16^, ^17^ and is based on the numb1er of studies with findings in the expected direction and their level of significance. For each study, we assigned a *z* value based on the level of statistical significance (*α*) and direction of association (expected direction of association based on research hypothesis vs. unexpected direction of association). If associations were in the expected direction, then *z* = 1.96 for *α* = 0.05, and *z* = 1.64 for *α* = 0.10; if associations were in the unexpected direction, then *z* = −1.96 for *α* = 0.05, and *z* = −1.64 for *α* = 0.10; *z* = 0.00 was assigned to null (statistically not significant) associations with *p* > 0.10. We summed the *z* value for each reported finding and weighted these by the quality assessment score for each study, divided by the square root of the sum of squared quality assessment scores. To determine the strength of association for each built–environment–outcome combination, a two‐tailed *p* value was computed for each weighted *z* value with interpretation of weak evidence if *p* < 0.05, strong evidence if *p* < 0.01, and very strong evidence if *p* < 0.001.^16^ To avoid overrepresentation of individual studies reporting built environment–outcome associations by different subgroups (e.g., boys/girls, geographic area, and age group), we applied fractional weights to each finding so that the sum of the weights across all reported associations was 1.^17^ For example, if a study reported a positive association of fine particulate matter with childhood obesity, but that association was significative (*α* = 0.05) only in boys (*z* = 1.96) and not in girls (*z* = 0.00), the *z* value assigned to the study was 1.96 ∗ 0.5 + 0 ∗ 0.5 = 0.98. Following the standard set for meta‐analysis, associations for each built environment feature–outcome combination were only synthesized if five or more studies reported such associations.

## 3 RESULTS

Results are presented separately for each built environment characteristics: (1) traffic noise, (2) air pollution, (3) neighborhood walkability, and (4) accessibility and availability of parks and playgrounds. PRISMA flow diagrams are shown in Figures S1–S4, respectively.

Our search initially identified 1192 studies with some studies included in more than one built environment domain. After the removal of duplicates and applying screening criteria, we included four studies on traffic noise and childhood obesity, 14 studies on air pollution, 19 studies on neighborhood walkability, and 28 studies on accessibility and availability of parks and playgrounds. Data extracted for all studies meeting eligibility criteria are presented in Tables S4a–S4d. We did not find evidence for publication bias (Figure S5).

### 3.1 Childhood obesity and traffic noise

#### 3.1.1 Study characteristics

The four studies investigating effects of traffic noise on childhood obesity were recent (2016–2019) longitudinal studies from Northern Europe (Table 1).^18^, ^19^, ^20^, ^21^ Two studies used national birth cohorts,^19^, ^20^ the others longitudinal studies with national coverage. Sample sizes ranged from 3963 to 40,974 participants. All studies assessed exposure to noise through standard modeling methods, linked to the home addresses of the subjects. Three studies used an implementation of the Nordic prediction method for road traffic noise, one study a national noise standard.^18^ Methodologies between studies were generally comparable. The Swedish study^20^ obtained height and weight from school and health records and, in part, measurements, whereas the three other studies used height and weight from questionnaires. The Norwegian study^21^ accounted for age and sex in the model via interaction terms to explore the effect of noise on BMI trajectory, whereas all others studies either used a age/sex standardization of BMI (BMI *z* score) and/or categorized BMI based on sex and age‐specific cut‐offs for overweight and obese from the International Obesity Task Force (IOTF). All studies accounted for age, sex, and maternal education in analysis, in addition to other study‐specific confounders including maternal BMI prior pregnancy,^19^, ^20^, ^21^ parental smoking,^18^, ^19^, ^20^ neighborhood socioeconomic status,^18^ and physical activity.^20^ One study controlled further for urbanization and nitrogen oxides (NO_x_).^19^ Studies used either linear mixed models,^18^, ^21^ multiple regression,^19^ or quantile regression^20^ with increasing levels of adjustment. All studies were of high quality with scores of 9–10 out of the maximum 12 stars (see Table S5).

**TABLE 1:** Summary of characteristics of articles

Noise (*n* = 4)	Publication year	2016 (1); 2018 (1); 2019 (2)
	Study design	Longitudinal (4)
	Country	Norway (1); Sweden (1); Denmark (1); Netherlands (1)
	Sample size	1000–5,000 (2); 5000–10,000 (1); 10,000–50,000 (1)
	Age group	Children 0–9 y (3); adolescents 10–18 y (2)
	Exposure model	Nordic prediction method (3); STAMINA (1)
	Outcome variables	BMI (4); overweight/obese classification (4)
Air pollution (*n* = 14)	Publication year	2014 (2); 2015 (1); 2017 (3); 2018 (3); 2019 (5)
	Study design	Cross‐sectional (4); longitudinal (11)
	Country	USA (7); UK (1); Italy (1); Spain (1); Sweden (1); Netherlands (1); Hong Kong (1); China (1)
	Sample size	<1000 (3); 1000–5000 (7); 5000–10,000 (2); 10,000–50,000 (2)
	Age group	Children 0–9 y (11); adolescents 10–18 y (8)
	Exposure variables	NO_x_ (11); PM_2.5_ (8); PM_10_ (5); SO_2_ (2); O_3_ (1)
	Outcome variables	BMI (7); overweight/obese classification (7); fat mass (2); waist‐to‐hip ratio (1)
Neighborhood walkability (*n* = 19)	Publication year	2007 (1); 2008 (1); 2011 (1); 2012 (1); 2013 (3); 2014 (1); 2015 (2); 2017 (1); 2018 (2); 2019 (5); 2020 (1)
	Study design	Cross‐sectional (15); longitudinal (5)
	Country	USA (12); Canada (3); Germany (1); UK (1); Spain (1); Israel (1)
	Sample size	<1000 (9); 5000–10,000 (1); 10,000–50,000 (9)
	Age group	Children 0–9 y (13); adolescents 10–18 y (15)
	Exposure variables	Walkability index (11); Walk Score (3); intersection density 1(8); residential density (6); land use mix (4)
	Outcome variables	BMI‐derived outcomes (BMI‐*z*, BMI trajectories, overweight, and obesity prevalence) (18); waist circumference (2); skinfold thickness (2); body fat % (1)
Availability and accessibility of parks and playgrounds (*n* = 28)	Publication year	2008 (1); 2009 (1); 2011 (3); 2012 (1); 2013 (2); 2014 (3); 2015 (5); 2016 (2); 2017 (4); 2018 (5); 2019 (2)
	Study design	Cross‐sectional (20); longitudinal (8)
	Country	USA (13); UK (4); Germany (2); Spain (2); Netherlands (1) Lithuania (1); Australia (2); Canada (2); Nepal (1)
	Sample size	<1000 (7); 1000–5000 (11); 5000–10,000 (4); 10,000–50,000 (6)
	Age group	Children 0–9 y (24); adolescents 10–18 y (21)
	Exposure variables	Distance (9); park area (11); number of parks (8); presence/absence of parks (6); NDVI (3)|Around home (26); Around school (2).|Playground specific (3)
	Outcome variables	BMI derived outcomes (including BMI‐*z*, BMI trajectories, BMI percentiles, and weight status) (33); waist circumference (1); waist‐to‐height ratio (1); sum of skinfold (1); body fat % (1)

Abbreviations: BMI, body mass index; NDVI, Normalized Difference Vegetation Index; PM, particulate matter; STAMINA, Standard Model Instrumentation for Noise Assessments; y, years.

#### 3.1.2 Summary of findings

Due to the small number of studies, meta‐analysis was not applied and findings descriptive. Impacts of traffic noise on childhood obesity were observed in three studies, but overall results were mixed and varied by life stage (see Table S4a). Positive associations of road‐traffic noise exposure during pregnancy and the risk of being with overweight in school‐age children (7/8 years) were observed in Denmark and Norway^19^, ^21^ but not Sweden.^20^ For the same age group, no impact of childhood noise exposure on weight was found.^18^, ^19^, ^20^, ^21^ Wallas et al., however, studied the effect of traffic noise exposure during adolescence and found a strong association with adolescence BMI between the ages of 8 and 16 years, which was slightly stronger for girls.^20^

### 3.2 Childhood obesity and air pollution

#### 3.2.1 Study characteristics

The majority (*n* = 11) of the 14 reviewed studies were longitudinal studies, the others cross‐sectional. Half of the eligible studies were conducted in the United States (*n* = 7), followed by European (*n* = 5) and Asian studies (*n* = 2). The largest sample size was 30,056 children in a cross‐sectional study.^22^ Longitudinal studies were smaller, also due to a loss to follow‐up.^23^ Most studies (*n* = 8) were conducted in urban settings, resulting in ~80% of participants residing in urban areas. Most studies (*n* = 9) focused on childhood, only three studies on adolescents.^18^, ^24^, ^25^ Studies analyzed a wide range of air pollutants in relation to childhood obesity. The most studied pollutant was particulate matter with diameter less than 2.5 μm (PM_2.5_) (*n* = 8), followed by NO_x_ (*n* = 7), nitrogen dioxide (NO_2_) (*n* = 6), PM_10_ (*n* = 5), sulfur dioxide (SO_2_) (*n* = 2), ozone (*n* = 1), and black carbon (*n* = 1). All studies assessed air pollution exposure at the home address, one study also at school.^26^ Five studies modeled air pollution exposure using dispersion models,^25^, ^27^, ^28^, ^29^, ^30^ five others used Land Use Regression.^18^, ^26^, ^31^, ^32^, ^33^ Two studies interpolated measurement data from multiple monitoring stations using inverse distance weighting^24^, ^34^ and two studies linked measurements from the nearest monitoring station.^22^, ^35^ BMI was used as main outcome in six studies,^24^, ^25^, ^27^, ^30^, ^31^, ^34^ two longitudinal studies used BMI trajectories,^30^, ^32^ and seven studies used weight status classification. Different growth charts and guidelines were used to standardize BMI to adjust for age and sex (BMI *z* score). The most common was the Centers for Disease Control and Prevention (CDC) growth chart, used in five U.S. studies^24^, ^25^, ^30^, ^31^, ^35^ and one study from China.^22^ Three studies used the World Health Organization (WHO) growth reference data and one the IOTF indications. Two studies utilized national standards from the United Kingdom^29^ and Sweden.^28^ The majority of studies adjusted for age and sex, one study used Tanner stage,^24^ and one only studied 4‐year‐old children.^28^ Three studies did not adjust for age but used age and sex standardized BMI measures.^26^, ^29^, ^35^ Covariates varied widely across studies and included parental socioeconomic status, maternal BMI, birth weight, parental smoking, and passive smoking exposure. All studies had a quality rating of good, ranging from 9 to 11 stars (see Table S5).

#### 3.2.2 Summary of findings

To synthesize findings using the *z* test, we combined NO_2_ and NO_x_ results, and PM_2.5_ and PM_10_ were considered separately (Table 2). No *z* statistics was derived for SO_2_, ozone, and black carbon due to the small number of studies. Of the 11 studies that looked at NO_2_/NO_x_, five reported significative associations with BMI‐derived outcomes,^18^, ^22^, ^24^, ^25^, ^27^ and four studies did not find significative results.^26^, ^28^, ^29^, ^33^ Two of the studies had mixed results, one found an effect only in boys^34^ and in one study the effect dependent on the exposure period.^30^ Overall, the association of NO_2_/NO_x_ exposure on childhood obesity was strong with a two‐tailed *p* value from the weighted *z* value being *p* = 0.003. Overall, there was no statistically significant effect of PM_2.5_ on childhood obesity with *p* = 0.10. Five out of the eight studies investigating PM_2.5_ did not find any significant effect, two showed a positive association,^24^, ^35^ and one found an effect only in boys.^31^ Only one of the five studies looking at PM_10_ reported an effect,^22^ reflected by the *p* value of 0.15. SO_2_ and ozone were associated with increased prevalence of obesity in one of the studies,^34^ but in another study, a higher SO_2_ in utero and in childhood was associated with lower BMI at ~13 and ~15 years.^22^ Four studies did not find any significant evidence of a link between air pollution and childhood obesity,^26^, ^28^, ^32^, ^33^ two of which were conducted in areas of modest level air pollution.^28^, ^32^

**TABLE 2:** Summary of findings

	Built environment characteristics	Number of studies for the specific exposure–outcome		Synthesis of findings	
		Total	Significant results	*z* score	*p* value
Air pollution	NO_2_/NO_x_	*n* = 11	*n* = 5.8 (53%)	3.422	**0.0006** ***
	PM_2.5_	*n* = 8	*n* = 2.5 (31%)	1.643	0.100
	PM_10_	*n* = 5	*n* = 1.5 (30%)	1.441	0.150
	SO_2_	*n* = 2	*n* = 1.75 (88%)	‐	
	O_3_	*n* = 1	*n* = 1 (100%)	‐	
Neighborhood walkability	Walkability index	*n* = 10	*n* = 1.8 (18%)	1.083	0.279
	Walk score	*n* = 2	*n* = 0.2 (10%)	‐	‐
	Walkable streets	*n* = 1	*n* = 1 (100%)	‐	‐
	Intersection density	*n* = 7	*n* = 3.6 (51%)	2.805	**0.005** **
	Residential density	*n* = 6	*n* = 1.3 (22%)	1.206	0.223
	Land use mix	*n* = 4	*n* = 1 (25%)	‐	‐
Availability and accessibility of parks and playgrounds	Distance to nearest park	*n* = 9	*n* = 2.2 (24%)	1.373	0.170
	Park area	*n* = 10	*n* = 4 (40%)	2.451	**0.014** *
	Number of parks	*n* = 8	*n* = 2 (25%)	1.448	0.148
	Presence/absence of parks	*n* = 5	*n* = 4 (80%)	3.498	**0.0005** ***
	Normalized Difference Vegetation Index (NDVI)	*n* = 3	*n* = 1 (33%)	‐	‐
	Playgrounds only (distance, area, presence)	*n* = 3	*n* = 0	‐	‐

*Note*: Total number of studies for each specific exposure–outcome combination (only BMI‐derived outcomes in this case), and the number reporting statistically significant associations, including fractional results to account for mixed findings (e.g., valid only in boys and not in girls = 0.5) with percentages in brackets. *z* scores and *p* values are results from the *z* test. Statistically significative *p* values are highlighted.

* *p* < 0.05—weak evidence.

** *p* < 0.01—strong evidence.

*** *p* < 0.001—very strong evidence

### 3.3 Childhood obesity and neighborhood walkability

#### 3.3.1 Study characteristics

Most of the 19 included studies used a cross‐sectional study design (*n* = 14), four were longitudinal, and one study included both a longitudinal and a cross‐sectional approach.^36^ Most studies were conducted in the United States (*n* = 12), three were conducted in Canada,^37^, ^38^, ^39^ and the others in Germany,^40^ the United Kingdom,^23^ Spain,^41^ and Israel.^42^ Four studies were based on large population samples (*n* > 35,000) reflecting the cross‐sectional study designs, and one was a longitudinal study conducted in the United States with a small loss during follow‐up.^43^ Six studies relied on medium sample sizes (9440 < *n* < 14,084) and the other nine on small sample sizes (*n* < 1000). Four studies were focused on children (<7 years old), six included only adolescents, and the nine studies included both categories. Among the included studies, several methodologies were used to quantify neighborhood walkability. The most common method (*n* = 10) was the walkability index based on the approach developed by Frank et al.^44^ The original method by Frank et al. incorporated land use mix, street connectivity, net residential density, and retail floor area ratios, giving street connectivity twice the weight of the other three variables. Often studies used modified versions of the walkability index, that is, giving street connectivity the same weight as the other variables, using destinations as proxy for land use, not accounting for the retail floor area ratio or including additional elements such as access to facilities and parks. The three main components of the walkability index (land use mix, street connectivity, and net residential density) were often individually analyzed. Two studies used the Walk Score,^37^, ^45^ a web‐based tool (www.walkscore.com) that relies mainly on the distance to various amenities and includes population density and road metrics such as block length and intersection density. One study adopted a different approach by deriving a walkability index composed of land use mix, sidewalks, sidewalk buffers, sidewalk/street lighting, other sidewalk elements, traffic lights, pedestrian signal at traffic lights, marked crosswalks, pedestrian crossing, and other signage and public transport.^46^ Except one study that analyzed percentage of body fat as outcome,^47^ all studies used BMI‐derived outcomes (BMI *z* score, BMI trajectories, overweight, and obesity prevalence), two of which analyzed waist circumference^39^ and skinfold thickness^48^ as additional measures. Sex was always considered as covariate, and age was missing only in one study.^49^ Other covariates relating to individual, household, and neighborhood confounders were included in the models. The most used were race/ethnicity, parental education, and neighborhood socioeconomic status. In general, studies were of good quality, with scores ranging from 8 to 11. The main factors that penalized some of the studies were small sample size, low representativeness of the general population, and the use of self‐reported data.^43^, ^46^, ^50^

#### 3.3.2 Summary of findings

There was limited evidence that the walkability index is linked to childhood obesity (*p* = 0.28), with only one out of 10 studies finding significant associations^40^ (Table 2). Two further studies showed mixed results based on sex (effect on bodyweight status in girls, but not boys)^38^ and geographic area (healthy BMI associated with higher levels of walkability in one of three studied cities).^42^ The Walk Score was associated with decreased BMI *z* score in rural but not urban youths in one study^45^ but did not show any significant association in another study.^37^ The walkability index based on street element characteristics, however, did identify a significant association with childhood obesity. With regards to individual walkability indicators, street intersection density was the most widely used indicator (*n* = 7). Three studies found significant associations with childhood obesity,^36^, ^43^, ^51^ one study found a weak positive association,^52^ mixed results were found in two studies, with effects observed in girls but not boys,^38^ and one out of three studied cities.^42^ The *z* test revealed strong evidence to support a link between street intersection density and obesity measures (*p* = 0.005). Out of six studies analyzing associations with population density, only one study found an effect of lower residential density being linked to higher BMI *z* score,^36^ and one study found an effect only in girls. Overall, the evidence did not suggest a link between population density and childhood obesity (*p* = 0.23). Land use mix was only analyzed in four studies, with one study finding a significant association.

### 3.4 Childhood obesity and accessibility and availability of parks and playgrounds

#### 3.4.1 Study characteristics

The dominant study design of the 28 included studies was cross‐sectional (*n* = 20), the others longitudinal (*n* = 8).^18^, ^29^, ^53^, ^54^, ^55^, ^56^, ^57^ One of the longitudinal studies conducted a quasi‐experiment, which considered a pre‐park and post‐park time frame and dividing the children into those who live near the park (the exposure group) and those who live further from the park (the control group) to examine how exposure to a newly built park translates to changes in BMI *z* score over time.^58^ Almost half of the studies were conducted in the United States (*n* = 13), 10 studies were conducted in Europe, four of which in the United Kingdom,^29^, ^56^, ^59^, ^60^ two studies from Germany^61^, ^62^ and Spain,^63^, ^64^ and one from the Netherlands^18^ and Lithuania.^65^ The sample sizes ranged from 93 to 41,283. Seven studies used small cohorts with less than 1000 subjects,^47^, ^48^, ^53^, ^64^, ^66^, ^67^, ^68^ most studies used medium size cohorts (*n* = 15) not exceeding 7000 participants, four studies included larger samples over 10,000 participants,^69^, ^70^, ^71^, ^72^ and two studies included very large samples of around 40,000 subjects.^52^, ^73^ Five studies considered a wide age range up to 18 years. Seven studies included children under the age of 9 years,^48^, ^59^, ^60^, ^62^, ^65^, ^70^, ^74^ and four studies included exclusively adolescents of at least 10 years.^61^, ^64^, ^73^, ^75^ Twelve studies included both children and adolescents with ages ranging from 4 to 18 years.

Most studies analyzed park accessibility and availability based on children's place of residence, and two studies focus on the school environment.^62^, ^72^ The definition of the sphere of influence was in 14 studies based on circular or network buffers ranging from 100 to 3000 m in radius from the pace of residence, one study that considered a 10‐mile (16,000 m) radius.^53^ Eight studies based their analysis on official administrative or statistical boundaries and three studies analyzed distance from the nearest park, without defining a sphere of influence.^52^, ^58^, ^64^ The remaining studies used neighborhood area without further specifications on the delimitations. The most used exposure metric was the relative amount of park surface in the sphere of influence (*n* = 11). Other studies quantified exposure through the dichotomous variable presence/absence of parks, the number or density of parks, and the distance from the nearest park. Four studies used the satellite‐derived Normalized Difference Vegetation Index (NDVI) to quantify the greenness of the surrounding environment. The definition of park/greenspace was inconsistent across studies. Most studies identified areas intended as urban free‐usable greenspace. Some studies identified specific features (e.g., children playgrounds), and others used a broad approach (e.g., NDVI), considering the total amount of vegetation without distinct function.

The outcomes analyzed were BMI *z* score, BMI trajectories, BMI percentiles, and weight status. Anthropometric measures were rarely used: waist circumference (*n* = 3),^55^, ^76^, ^77^ waist‐to‐height ratio (*n* = 1),^55^ sum of skinfold (*n* = 1),^48^ and percentage body fat (*n* = 2).^47^, ^77^

The quality of the studies was either fair (*n* = 6) or good (*n* = 22). The main reasons for fair quality were small sample sizes, self‐reported outcomes (height and weight), or study population scarcely representative of the population (see Table S5).

#### 3.4.2 Summary of findings

Due to the great variability in exposure metrics, we synthesized findings across the following exposure categories: distance to the nearest park (*n* = 9), park area (*n* = 10), number of parks (*n* = 8), and presence/absence of parks (*n* = 5). Only three studies analyzed NDVI, which was insufficient for meta‐analysis according to our criteria (Table 2). The *z* test and related *p* value suggest that there was insufficient evidence to support an association of distance to park and childhood obesity (*p* = 0.170). Out of the nine studies, only one found a significant association.^67^ Two studies concluded with mixed findings: One study found a significant association in boys of all ages and girls of high school age but not in younger girls,^52^ and one study found an significant association in children living in urban areas but not those in rural areas.^18^ The *p* value suggested weak evidence of an association with percentage of park area (*p* = 0.014). Three studies found significant associations, six studies found no statistically significant effects, and two studies had mixed results, with effects only found in boys and older children. The *p* value showed little evidence of an effect of number or density of parks on childhood obesity (*p* = 0.148). One study found a significant association, five studies did not find significant associations, and one study reported mixed results with effects only in girls.^69^ The intervention study did, however, find an effect in the intervention group, which could not be replicated in the control group.^53^ We identified strong evidence on the presence of a park within the sphere of influence and childhood obesity (*p* < 0.001). Out of the five studies, four studies found statistically significant effects. Results from the three studies that explored the effect of greenness via the NDVI suggest a potential association in the more proximal environment of less than 250 m.^18^, ^63^, ^65^ Three studies specifically focused on playgrounds, and none of them found statistically significative associations.

## 4 DISCUSSION

### 4.1 Impact of built environment characteristics on childhood obesity

We systematically reviewed the ep1idemiological evidence on the influence of four built environment characteristics on obesity outcomes in children: traffic noise, air pollution, neighborhood walkability, and accessibility and availability of parks and playgrounds. To our knowledge, this is the first systematic review on this topic that applied a systematic synthesize of findings to evaluate the strength of the available evidence.

Studies were generally of high quality, using objectively measured outcome and exposure measures and adjusting for relevant confounders. Some studies, however, had small sample sizes, which were not necessarily representative of the overall population. Overall, 42% of studies used longitudinal data; however, the small number of longitudinal studies investigating effects of neighborhood walkability and parks accessibility should be emphasized.

We found very strong evidence of association of BMI‐derived obesity outcomes with NO_2_/NOx (*p* < 0.001) and presence/absence of parks in the neighborhood (*p* < 0.001), strong evidence with intersection density (*p* < 0.01), and some evidence with the amount of park area in the neighborhood (*p* < 0.05). There was little evidence of an effect on childhood obesity in relation to PM_2.5_, PM_10_, walkability index, residential density, distance to the nearest park, number of parks, and access to playgrounds.

Air pollution has been shown to decrease birth weight^78^ and might independently affect weight in childhood through epigenetic and behavioral adaptation. Some hypotheses on the mechanism involved in the exposure both during pregnancy and childhood were highlighted in previous publications: Prenatal growth restrictions can lead to growth spurts in early childhood with implications on increased weight into later childhood and adolescence^79^; heavy traffic roads, an important sources of air pollution, might deter active transport and reduce physical activity.^80^ Our findings point towards this direction with traffic‐related air pollutants NO_2_ and NO_x_ having a strong impact on increased weight in childhood, but not particulate matter (PM_2.5_ and PM_10_), which is driven to a lesser degree by local traffic.^18^ Another explanation could be the biochemical mechanism that emphasizes the role of NO_2_ as active oxidant involved in many physiological pathways in the human body, which might impact consequently the onset of obesity.^81^

Despite evidence suggesting a link between walkability and physical activity,^82^ we found little evidence of neighborhood walkability decreasing BMI‐derived outcomes. Intersection density is the only indicator of walkability that showed strong evidence of a negative association with childhood obesity. The central role of this measure in the walkability index has already been highlighted in the original equation by Frank et al., which gave street connectivity twice the weight of the other variables. Given the same source, road traffic, future studies should explore the effect of collinearity between the walkability components and other traffic‐related factors such as traffic noise and air pollution. Studies United States with only small number of studies from Europe North American cities have a different urban structure compared to European cities and results might not be directly comparable and transferable." The meaning of this sentence is not clear; please rewrite or confirm that the sentence is correct."?]‐‐> on walkability were mainly conducted in the United States with only small number of studies from Europe North American cities have a different urban structure compared to European cities and results might not be directly comparable and transferable. This should be explored further in future studies.

We also found strong evidence for the presence (or accessibility) of parks with decreased prevalence of childhood obesity, whereas studies focusing on playgrounds did not find significative associations. This is supported by findings from Bird et al. who concluded that parks that emphasize unstructured activities (i.e., with few team sport installations) were associated with lower percentage of truncal fat among children at risk of being with obesity.^83^

### 4.2 Methodological considerations

Some of the included studies investigated more than one built environment characteristics. Several studies explored walkability and parks.^29^, ^47^, ^48^, ^52^, ^70^, ^84^ Among the studies that considered walkability and greenspaces, walkability was not statistically significant, except intersection density in boys in one of the studies,^47^ and greenspace was at least partially associated with weight outcomes in all studies. No multi‐exposure interactions were evaluated in these studies, except for a Pearson correlation coefficient between intersection density and park space, which did not show collinearity.^47^ Overall, we found a lack of studies that explore the interaction between multiple exposures on childhood obesity. Bloemsma et al.^18^ investigated the combined effect of noise, air pollution, and park accessibility. They found that the association of NO_2_ with overweight remained after adjustment for noise and greenspace, but the associations between greenspace and overweight weakened substantially after adjustment for NO_2_, indicating that NO_2_ is driving the relationship. To better understand the complex relationship of multiple built environment characteristics on childhood obesity, more evidence is required.

Our review highlighted a strong presence of effect modifiers. Sex was the most studied effect modifier, but there was no consistency across studies. Two studies reported an increased effect in boys for the association between air pollution exposure and BMI,^31^, ^34^ but one of the studies found also an opposite effect considering waist‐to‐hip ratio as anthropometric measure, which was statistically significant only in girls.^31^ Walkability and intersection density were found to be associated with body weight status in girls but not in boys in one of the studies,^38^ but in another study, a high level of street connectivity was related to lower percentage of body fat only in boys.^47^ The association between park accessibility and obesity was gender‐dependent in five studies, of which three showed more significant effects on boys^52^, ^54^, ^55^ and two on girls.^47^, ^69^ Overall, sex affected the results in nine studies, concluding with an increased effect in boys in five studies, in girls in three studies, and with opposite effects depending on the considered anthropometric measure in one of the studies. Age was another common effect modifier, showing differential results in five studies. In one study, the exposure to road traffic noise was associated with increased BMI from school age to adolescence, but not at earlier ages, the relation increased in the older age groups.^20^ Age also modified the association between greenspace exposure and BMI in four studies (two of them were based on the same sample), always with increased effects in older children.^29^, ^52^, ^54^, ^55^ Another effect modifier was urbanization, with one study finding a negative association between walk score and BMI *z* score for youths in rural settings and a positive association among urban youths,^45^ whereas in another study, children living in a urban area had a negative association of the distance to the nearest park with weight status and no association for those living in rural areas.^18^ No studies analyzed effect modification by socioeconomic status, an important omission that could potentially highlight important pathways to health inequalities.

This systematic review assessed the strength of the evidence and identified the role of different elements of the built environment on childhood obesity, consolidated associations, and indicating areas in need of further evidence. Our review has some limitations. Due to the observational nature of included studies, no direct causal relationships can be inferred from the results. The absence of sample size restriction in the selection of studies allowed the inclusion of very small cohorts with results potentially not being transferable beyond the specific setting. The fact that some of the studies used self‐reported outcomes (weight and height) could also influence the quality of the results due to the introduction of error and bias in the outcome measures. Finally, it was not possible to conduct a meta‐analysis due to the large heterogeneity in study results, which could have influenced the validity of our findings. Previous reviews on the effect of the physical and built environment on childhood obesity, however, expressed the results only through descriptive synthesis or narrative review. The use of the *z* test is a strength that allows us to assess and quantify the strength of the associations.

## 5 CONCLUSION

In summary, we found strong evidence for an association of traffic‐related air pollution (nitrogen dioxide and nitrogen oxides exposure, *p* < 0.001) and built environment characteristics supportive of walking (street intersection density, *p* < 0.01 and access to parks, *p* < 0.001) with childhood obesity. Studies on traffic noise had mixed results and were too few to be included in the *z* test analysis. Future studies should consider the interactions between different environmental exposures and verify the role of age and sex as an effect modifier.

## CONFLICT OF INTEREST

The authors declare that they have no conflicts of interest.

## Supporting information

Table S1. Search Strategy
Table S2. Inclusion and exclusion criteria used to identify eligible studies
Table S3. Newcastle‐Ottawa Scale used for quality assessment, adapted for observational studies
Table S4a. Characteristics of studies included in the systematic review on traffic noise
Table S4b. Characteristics of studies included in the systematic review on air pollution
Table S4c. Characteristics of studies included in the systematic review on neighbourhood walkability
Table S4d. Characteristics of studies included in the systematic review on accessibility and availability of parks and playgrounds
Table S5. Quality assessment scores using modified Newcastle‐Ottawa Scale for studies on traffic noise
Figure S1. PRISMA flow diagram for traffic noise and childhood obesity
Figure S2. PRISMA flow diagram for air pollution and childhood obesity
Figure S3. PRISMA flow diagram for neighbourhood walkability and childhood obesity
Figure S4. PRISMA flow diagram for availability and accessibility of parks and playgrounds and childhood obesity
Figure S5. Publication bias by built environment characteristics
