# Supplementary material for: The built environment as determinant of childhood obesity: A systematic literature review
Source: Obes Rev. 2021 Dec 3;23(Suppl 1):e13385. doi: 10.1111/obr.13385 (PMC11475329; doi:10.1111/obr.13385)
Supplement: Supplementary file 1 — Table S1. Search Strategy Table S2. Inclusion and exclusion criteria used to identify eligible studies Table S3. Newcastle‐Ottawa Scale used for quality assessment, adapted for observational studies Table S4a. Characteristics of studies included in the systematic review on traffic noise Table S4b. Characteristics of studies included in the systematic review on air pollution Table S4c. Characteristics of studies included in the systematic review on neighbourhood walkability Table S4d. Characteristics of studies included in the systematic review on accessibility and availability of parks and playgrounds Table S5. Quality assessment scores using modified Newcastle‐Ottawa Scale for studies on traffic noise Figure S1. PRISMA flow diagram for traffic noise and childhood obesity Figure S2. PRISMA flow diagram for air pollution and childhood obesity Figure S3. PRISMA flow diagram for neighbourhood walkability and childhood obesity Figure S4. PRISMA flow diagram for availability and accessibility of parks and playgrounds and childhood obesity Figure S5. Publication bias by built environment characteristics [file OBR-23-e13385-s001.docx]

# SUPPORTING INFORMATION

**The built environment as determinant of childhood obesity: a systematic literature review**

Diego Malacarne^1^, Evangelos Handakas^1^, Oliver Robinson^1^, Elisa Pineda^2^, Marc Saez^3,4^, Leda Chatzi^5^, Daniela Fecht^1*^

*1 MRC Centre for Environment and Health, School of Public Health, Imperial College London, London, UK*

*2 Centre for Health Economics & Policy Innovation (CHEPI), Imperial College Business School, and School of Public Health, Imperial College London, London, UK*

*3 Research Group on Statistics, Econometrics and Health (GRECS), University of Girona, Spain*

*4 CIBER of Epidemiology and Public Health (CIBERESP), Madrid, Spain*

*5 Keck School of Medicine, University of Southern California, US*

**Content**

| Table S1. Search Strategy | Page 2 |
| --- | --- |
| Table S2. Inclusion and exclusion criteria used to identify eligible studies | Page 3 |
| Table S3. Newcastle-Ottawa Scale used for quality assessment, adapted for observational studies | Page 4 |
| Table S4a. Characteristics of studies included in the systematic review on traffic noise | Page 5 |
| Table S4b. Characteristics of studies included in the systematic review on air pollution | Page 7 |
| Table S4c. Characteristics of studies included in the systematic review on neighbourhood walkability | Page 11 |
| Table S4d. Characteristics of studies included in the systematic review on accessibility and availability of parks and playgrounds | Page 16 |
| Table S5. Quality assessment scores using modified Newcastle-Ottawa Scale for studies on traffic noise | Page 22 |
| Figure S1. PRISMA flow diagram for traffic noise and childhood obesity | Page 24 |
| Figure S2. PRISMA flow diagram for air pollution and childhood obesity | Page 25 |
| Figure S3. PRISMA flow diagram for neighbourhood walkability and childhood obesity | Page 26 |
| Figure S4. PRISMA flow diagram for availability and accessibility of parks and playgrounds and childhood obesity | Page 27 |
| Figure S5 Figure S5. Publication bias by built environment characteristics | Page 28 |

**Table S1. Search Strategy**

| **EMBASE/MEDLINE** | |
| --- | --- |
| Num. | Searches |
| 1 | Childhood Obesity/ Pediatric Obesity/ |
| 2 | ((child* or adolescent* or infant* or youth or pediatric) and (obesity or adiposity or overweight or overfat)).ti,ab,kw. |
| 3 | 1 or 2 |
| 4 | <Exposure> ^ƚ^ |
| 5 | <Exposure/obesity link>^ǂ^ |
| 6 | 4 or 5 |
| 7 | 3 and 6 |
| 8 | limit 7 to English language |
| 9 | limit 8 to humans |
| **WEB OF SCIENCE** | |
| AB=((child OR adolescent OR infant OR youth OR pediatric) AND (obesity OR adiposity OR overweight OR overfat) AND (noise)) OR  AB=((child OR adolescent OR infant OR youth OR pediatric) AND (obesity OR adiposity OR overweight OR overfat) AND ("Air pollution" or "particulate matter" or pm10 or "pm2.5" or co2 or "Carbon dioxide" or "Carbon monoxide")) OR  AB=((child OR adolescent OR infant OR youth OR pediatric) AND (obesity OR adiposity OR overweight OR overfat) AND (walkability)) OR  AB=((child OR adolescent OR infant OR youth OR pediatric) AND (obesity OR adiposity OR overweight OR overfat) AND (park OR "green space” OR "green spaces”)) OR  TI=((child OR adolescent OR infant OR youth OR pediatric) AND (obesity OR adiposity OR overweight OR overfat) AND (noise)) OR  TI=((child OR adolescent OR infant OR youth OR pediatric) AND (obesity OR adiposity OR overweight OR overfat) AND ("Air pollution" or "particulate matter" or pm10 or "pm2.5" or co2 or "Carbon dioxide" or "Carbon monoxide")) OR  TI=((child OR adolescent OR infant OR youth OR pediatric) AND (obesity OR adiposity OR overweight OR overfat) AND (walkability)) OR  TI=((child OR adolescent OR infant OR youth OR pediatric) AND (obesity OR adiposity OR overweight OR overfat) AND (park OR "green space” OR "green spaces”)) OR AK=((child OR adolescent OR infant OR youth OR pediatric) AND (obesity OR adiposity OR overweight OR overfat) AND (noise))  AK=((child OR adolescent OR infant OR youth OR pediatric) AND (obesity OR adiposity OR overweight OR overfat) AND ("Air pollution" or "particulate matter" or pm10 or "pm2.5" or co2 or "Carbon dioxide" or "Carbon monoxide"))  AK=((child OR adolescent OR infant OR youth OR pediatric) AND (obesity OR adiposity OR overweight OR overfat) AND (walkability))  AK=((child OR adolescent OR infant OR youth OR pediatric) AND (obesity OR adiposity OR overweight OR overfat) AND (park OR "green space” OR "green spaces”)) | |

* = truncation character (wildcard)

ti.ab. = limit to title, abstract fields and key word

^ƚ^<Exposure>: Noise/ or Noise Pollution; Air Pollution/; (Environment Design/ or Residence Characteristics/) and Walking/; recreational park/

^ǂ^<<Exposure/obesity link>: noise.ti,ab,kw.; (Air pollution or particulate matter or pm10 or "pm2.5" or co2 or Carbon dioxide or Carbon monoxide).ti,ab,kw.; walkability.ti,ab,kw.; (park* or green space*).ti,ab,kw.

**Table S2. Inclusion and exclusion criteria used to identify eligible studies**

| **Factor** | **Inclusion criteria** | **Exclusion criteria** |
| --- | --- | --- |
| Time of publication | Inception to May 2020 | None |
| Study subjects | Human, children and/or adolescents aged 18 years or under | Studies focused on animal subjects or human adults over the age of 18 years |
| Study area | Any geographical region, nation, or city | None |
| Outcome | Body weight status as heigh and weight, body mass index (BMI) or BMI standardised for age and sex (BMI *z*-score); features of the built environment and their association expressed by effect measure (e.g. odds ratio, relative risk) and confidence intervals | Studies that incorporated no outcome pertaining to weight status, features of the built environment and their association |
| Study design | Observational studies: cross‐sectional studies and longitudinal studies including prospective and retrospective cohort studies | Controlled experiments conducted in manipulated settings |
| Article type | Peer-reviewed journal articles | Letters, editorials, study or review protocols, or review articles |
| Language | English | Other than English |
| Exposure | Characteristics of the built environment must be the results of geospatial analysis (e.g. using a geographic information system) or models which use objective data and not based on questionnaires or self-reported data on the perceived exposure. For air pollution, only long-term exposure. | Studies based exclusively on questionnaires or self-reported data on the perceived exposure instead of objective data. For air pollution, short-term exposures. |

**Table S3. Newcastle-Ottawa Scale used for quality assessment, adapted for observational studies**

| **Selection** | S1 | Representativeness of the exposed population | Truly representative of the children and adolescent in the community (the whole population in the community) | ** |
| --- | --- | --- | --- | --- |
|  |  |  | Somewhat representative of the average in children and adolescent (subsample) | * |
|  |  |  | Selected group of children and adolescents (e.g. children in care) or no description of the study population |  |
|  | S2 | Selection of the non-exposed population | Drawn from the same community as the exposed population or | * |
|  |  |  | Drawn from a different community as the exposed population or no description of the derivation of the non-exposed population |  |
|  | S3 | Ascertainment of the exposure (risk factor) | Objectively defined exposure (e.g. data linkage and GIS). | ** |
|  |  |  | Self-reported exposure | * |
|  |  |  | No description of the exposure assessment tool. |  |
|  | S4 | Sample size | >10000 | ** |
|  |  |  | <10000 and >1000 | * |
|  |  |  | <1000 |  |
| **Comparability** | C1 | The subjects in different outcome groups are comparable, based on the study design or analysis. Confounding factors are controlled. | The study controls for the most important factor (age and sex) or just sex where there are no differences in age | * |
|  |  |  | The study controls for any additional factor (e.g. socioeconomic status). | * |
| **Outcome** | O1 | Assessment of the outcome | Independent blind assessment. | ** |
|  |  |  | Record linkage | ** |
|  |  |  | Self report | * |
|  |  |  | No description |  |
|  | O2 | Statistical test | Clearly described and appropriate, association, including confidence intervals and the probability level (p value). | * |
|  |  |  | Not appropriate, not described or incomplete. |  |

**Table S4a. Characteristics of studies included in the systematic review on traffic noise**

| First Author, Year | Study design | Country | Sample size | Age (years) | Exposure | Outcome | Confounders | Main findings | QA |
| --- | --- | --- | --- | --- | --- | --- | --- | --- | --- |
| Bloemsma, 2019^18^ | longit. | Netherlands | 3,680 | 3-17 | Standard Model Instrumentation for Noise Assessments (STAMINA) at follow-up address | Questionnaire data on height and weight, BMI categorised based on age-sex specific IOTF cut-offs | Age, sex, maternal and paternal level of education, maternal smoking during pregnancy, parental smoking in the child's, home and neighborhood socioeconomic status, region | No association | 9 |
| Christesen, 2016^19^ | longit. | Denmark | 40,974 | 7 | Nordic Prediction Method at residential addresses during i) pregnancy, ii) 7-year follow-up | BMI *z*-score (height and weight from questionnaire) | Maternal BMI prior to pregnancy, maternal smoking, maternal age at delivery, parity at birth, highest level of attained education, vocational training, higher education, disposable income, urbanization, NOx | Childhood exposure to road-traffic noise associated with a higher risk for childhood overweight, but not prenatal exposure. No associations between road traffic noise and BMI z-scores. No associations between railway noise and adiposity. | 10 |
| Wallas, 2019^20^ | longit. | Sweden | 4,089 | 2 months, 1, 2, 4, 8, 12, 16 | Nordic Prediction Method at residential addresses, pre- and postnatal exposure | Overweight and obesity based on age-sex specific IOTF cut-offs | Stratified by age and sex. Covariates: physical activity, maternal occupational noise exposure during pregnancy, parental smoking during infancy, maternal BMI during pregnancy, parental occupation, municipality at birth | Exposure to prenatal road-traffic noise not associated with BMI at birth through to adolescence. Association of childhood road-traffic noise exposure and increased BMI from school age to adolescence, but not pre-school age. | 10 |
| Weyde, 2018^21^ | longit. | Norway | 6,403 | 0.5, 1.5 3, 5, 7, 8 | Nordic Prediction Method at residential addresses at 6 time points from pregnancy to age 8 years | BMI (height and weight from questionnaire) | Age, sex, proximity to city center, BMI at birth, ethnicity, maternal education, mother's BMI before pregnancy | Association of road traffic noise during pregnancy and BMI trajectories from birth to age 8 years. No association for childhood exposure and BMI trajectories from age 18 months to 8 years | 9 |

longit: longitudinal study design, QA: quality assessment. References relate to list of references in the main manuscript.

**Table S4b. Characteristics of studies included in the systematic review on air pollution**

| First Author, Year | Study design | Country | Sample size | Age (years) | Exposure | Outcome | Confounders | Main findings | QA |
| --- | --- | --- | --- | --- | --- | --- | --- | --- | --- |
| Alderete, 2017^24^ | longit. | USA | 314 | 8–15, 18 | Inverse distance weighting of monitored concentrations within 50km; monthly exposure data for up to 12 months prior to each visit. Pollutants: NO_2_, PM_2.5_ | BMI, body fat percent, BMI percentile and *z*-score (derived from CDC age- and sex-specific growth charts) | Tanner stage, year of study entry. Participant-specific intercepts were included as a function of sex, social position category, study wave, and a participant-level random intercept. | Higher NO_2_ and PM_2.5_ exposures associated with more rapid increases in BMI and central adiposity in children who were already overweight and obese at study entry. | 9 |
| Bloemsma, 2019^18^ | longit. | Netherland | 3,680 | 3-17 | LUR model (ESCAPE) at follow-up address. Pollutants: NO_2_, PM_2.5_, PM_10_ and PM_2.5_ absorbance | BMI (weight and height derived from the parental questionnaires), overweight based on age-sex specific IOTF cut-offs | Age, sex, maternal and paternal level of education, maternal smoking during pregnancy, parental smoking in the child's, home and neighborhood socioeconomic status, region | Increased odds of being overweight with increasing exposure to NO_2_ and PM_2.5 absorbance_, but not with to PM_2.5_ and PM_10_ | 9 |
| Chiu, 2017^31^ | longit. | USA | 239 | 4 | Hybrid LUR using aerosol optical depth; prenatal exposure at place residence  Pollutants: PM2.5 | BMI *z*-scores based on age-sex specific CDC growth charts, body fat, waist and hip circumferences | Maternal age, race/ethnicity, educational status, sex, gestational age at birth, and birth weight, mother’s pre-pregnancy BMI | Increased prenatal PM_2.5_ exposure strongly associated with increased body size in boys and with an indicator of body shape in girls. | 9 |
| De Bont, 2019^26^ | cross - sect. | Spain | 2,660 | 7–10 | LUR model (ESCAPE) at residential address, 1-year prior to study.  Pollutants: NO_2_, NOx, PM_10_, PM_2.5_, PM_Coarse_ and PMabsorbance_._  Measured levels of NO_2_, PM_2.5_, elemental carbon and ultrafine particles in schoolyards for two 1-week campaigns | BMI *z*-scores based on age-sex specific WHO growth reference, 2007 | Parental education, employment status, and country of birth, maternal smoking during pregnancy, adopting status, exposure to environmental tobacco smoke, number of siblings, physical activity | Exposure to NO_2_, PM_2.5_ and elemental carbon at schools associated with higher odds of overweight or obese at medium compared to low levels of exposure. PM_10_ exposure at associated with a 10% increase in the odds of being overweight or obese. Associations for BMI z-score similar in direction but not statistically significant | 10 |
| Dong, 2014^22^ | cross - sect. | China | 30,056 | 2-14 | Measurements from municipal air pollution monitoring stations obtained four to one year before recruitment.  Pollutants: PM_10_, SO_2_, NO_2_, and ozone | BMI, categorized based on age-sex specific CDC growth charts | Age, sex, breast feeding, low birth weight, parental education, area of residence, closeness to main road, home decoration, home cola use, ventilation device in kitchen, air exchange in winter, house pets, passive tobacco smoking exposure. | Exposure to high concentrations of ambient air pollution positively associated with overweight and obesity among children of 2-13 years of age | 11 |
| Fioravanti, 2018^33^ | cross - sect. and longit. | Italy | 719, follow-ups: 581, 499 | 4, 8 | LUR model (ESCAPE) estimated between birth and the first four years of life.  Pollutants: NO_2_, NOx, PM_10_, PM_2.5_, PM_Coarse_ and PM2.5absorbance | BMI *z*-score based on age-sex specific WHO growth reference, waist circumference and waist-to-hip ratio | Age, sex, maternal education, paternal education, maternal pre-pregnancy BMI, maternal smoking during pregnancy, maternal age at delivery, gestational age, child birth-weight, breastfeeding duration, age at weaning | No association of vehicular traffic with being with overweight/obesity at 4 or 8 years of age | 9 |
| Fleisch, 2019^32^ | longit. | USA | 1,649 | 0.5, 3, 7 | Aerosol optical depth estimated during the third trimester of pregnancy. Pollutants: PM_2.5_; black carbon | BMI, BMI trajectories | Maternal race/ethnicity, education, parity, and smoking habits, sex, birth weight, length of gestation, income | No association of prenatal exposure to traffic pollution on BMI trajectory from birth through mid-childhood | 10 |
| Frondelius, 2018^28^ | cross - sect. | Sweden | 5,815 | 4 | Dispersion model (AERMOD), prenatal exposure.  Pollutants: NOx | Obesity and overweight based on BMI adjusted for Swedish children | Parity, maternal and paternal BMI, socioeconomic status, smoking during pregnancy, exposure to passive smoking, breastfeeding, and sex | No associations of foetal exposures to elevated levels of NOx with increased risk for childhood obesity | 10 |
| Huang, 2019^34^ | longitudinal | Hong Kong | At the four follow-ups: 6,616, 6,462, 5,580, 4,190 | 9, 11, 13, 15 | Inverse distance weighting of mean monthly monitored concentrations at different growth phases (in utero, in infancy, and in childhood).  Pollutants: PM_10_, SO_2_, NO, NO_2_) | BMI | Neighborhood income, household income, mother’s migration status, highest parental education, sex, age at measurement, maternal age at birth, parity, maternal smoking | Among boys, associations of higher SO_2_ in utero with lower BMI at ~13 and ~15 years, higher SO_2_ in childhood with lower BMI at ~15 years, and higher NO_2_ in childhood with higher BMI at ~9, ~13, and ~15 years | 10 |
| Jerret, 2014^27^ | longit. | USA | 4,257 | 05-11 | Dispersion model (CALINE4) at baseline address.  Pollutants: NOx | BMI | Age, sex, town, race/ethnicity, poverty, crime rate and others (>50 confounding variables at the individual, neighborhood, school, and community level) | Traffic-related air pollution associated with obesity. Traffic-related air pollution had significant effect on BMI growth and BMI level at age 10. | 10 |
| Kim, 2018^30^ | longit. | USA | 2,318 | 1,2,3,4, 10 | Dispersion model (CALINE4) in utero and first year of life.  Pollutants: NOx | BMI trajectories at 4 yrs and BMI at 10 yrs, overweight and obese based on age-sex specific CDC growth charts | Age, sex, race/ethnicity, parental education | Increased first year of life near-road freeway NOx exposures associated with increased velocity of childhood BMI growth trajectory and higher attained BMI at 10 years. Increased childhood near-roadway exposures from non-freeway sources associated with increased BMI growth and higher BMI at 10 years | 9 |
| Mao, 2017^35^ | longit. | USA | 1,446 | 02-8 | Measurement from nearest monitor station during pregnancy and the first 2 y of life.  Pollutant: PM_2.5_ | BMI *z*-score based on age-sex specific CDC growth charts | Maternal age at delivery, race/ethnicity, education level, smoking status during pregnancy, diabetes, marital status, household income per year, maternal pregnancy BMI, season of delivery, preterm birth, birth weight, and breastfeeding | Positive association of PM_2.5_ in utero and from birth through year 2 | 10 |
| McConnel, 2015^25^ | longit. | USA | 3,318 | 10-18 | Dispersion model (CALINE4) at study entry.  Pollutants: NOx | BMI, overweight and obesity categorized based on age-sex specific CDC growth charts, sex-specific BMI trajectories | Ethnicity, age, race, sex, year of enrolment, community | Air pollution contribute to development of childhood obesity | 10 |
| Wilding, 2019^23^ | longit. | UK | 14,084 and 5,637 at the two follow-ups | 4, 5, 10, 11 | Dispersion model, annually.  Pollutants: PM_2.5_, PM_10_, NOx | BMI converted to age-sex specific centiles, according to 1990 UK weight reference | Maternal BMI, age, education, ethnicity, smoking at the start of pregnancy and parity | PM_10_ positively associated with overweight/obesity (at lower geographical level, but not neighbourhood level), but not among children who moved. | 11 |

longit: longitudinal study design, QA: quality assessment. References relate to list of references in the main manuscript.

**Table S4c. Characteristics of studies included in the systematic review on neighbourhood walkability**

| First Author, Year | Study design | Country | Sample size | Age (years) | Exposure | Outcome | Confounders | Main findings | QA |
| --- | --- | --- | --- | --- | --- | --- | --- | --- | --- |
| Colley, 2019^39^ | cross-sectional | Canada | 10,852 (including adults) | 3-17 | Walkability index: intersection density, dwelling density, points of interest. | BMI, waist circumference, weight status based on age-sex specific WHO growth reference | Age, sex, household income and household education | Walkability not associated with BMI and waist circumference in children | 11 |
| Duncan, 2014^36^ | cross-sectional longit. | USA | 49,770 and 46,813 at the follow ups | 4-18 | Separate walkability indicators: residential density, traffic density, average speed limit, sidewalk completeness, intersection density, land use mix | BMI *z*-score (cross-sectional) change in BMI *z*-score (longit) | Age, sex, race/ethnicity, neighborhood median household income | Living in neighborhoods with fewer recreational open spaces and less residential density, traffic density, sidewalk completeness, and intersection density associated with higher cross-sectional BMI *z*-score and increase in BMI *z*-score over time. | 11 |
| Gose, 2013^40^ | longit. | Germany | 485 | 5-7, 9-11 at the follow ups | Walkability index: population density, road connectivity, land-use mix | Age-sex specific German BMI percentile | Sex, maternal weight, maternal educational, nationality of the family | Walkability inversely associated with children’s BMI | 9 |
| Hagani, 2019^42^ | cross-sectional | Israel | 904 | 15-18 | Walkability index: residential density, intersection density, land-use mix | BMI | Age, area socioeconomic status, physical activity, sedentary behaviour and nutritional home environment | Healthy BMI associated with higher levels of walkability in one city, no significative results for the other two cities | 9 |
| Hsieh, 2015^47^ | cross-sectional | USA | 576 | 8-18 | Number of street intersections per square mile | Percent body fat, BMI *z*-score, waist circumference. BMI *z*-score based on age-sex specific CDC growth charts | Age, Tanner stage, neighborhood socio-cultural characteristics, spatial autocorrelation | Higher street connectivity associated with lower % body fat in boys | 8 |
| Jia, 2019^51^ | longit. | USA | 9440 | 6-14 | Street intersection density, residential density, fitness facility density and recreational facility density | BMI, overweight based on age-sex specific CDC growth charts | Age, sex, race/ethnicity, socioeconomic status, parental educational level and urbanicity | Intersection density in 1998–2007 associated with lower BMI and lower obesity risk in 2007. Girls and boys who lived more densely populated neighborhoods (but not highest) in 1998 had lower risk of overweight and obesity in 2007. | 10 |
| Kligerman, 2007^49^ | cross-sectional | USA | 98 | 14-17 | Walkability index: land use mix, retail density, street connectivity, residential density | BMI | Sex, ethnicity | No statistically significant association | 9 |
| Lovasi, 2011^48^ | cross-sectional | USA | 428 | 2–5 | Separate walkability indicators: population density, land use mix, density of subway, density of bus stop, intersection density | Skinfold thicknesses; BMI *z*-score based on age-sex specific CDC growth charts, bodyweight status. | Age, sex, race/ethnicity, mother’s age, birth outside of the USA, Spanish language, employment status, number of rooms in the home, neighborhood characteristics | Subway stop density associated with adiposity | 8 |
| Lovasi, 2013^70^ | cross-sectional | USA | 11,562 | 3-5 | Walkability index: residential density, land use mix, retail floor area ratio, intersection density, subway stop density | BMI *z*-score based on age-sex specific CDC growth charts | Age, sex, race/ethnicity, neighborhood characteristics | No statistically significant association | 10 |
| Molina-Garcia, 2017^41^ | cross-sectional | Spain | 325 | 14–18 | Walkability index: intersection density, net residential density, land use mix | BMI, BMI percentile based on age-sex specific CDC growth charts | Age, sex, days per week at primary address, distance to school, driver license; household number of children <18 years old, number of motor vehicles, number of years current address, workout equipment, spatial clustering | No statistically significant association | 9 |
| Saelens, 2012^84^ | cross-sectional | USA | 730 | 6–11 | Walkability index: residential density, retail floor area ratio, land-use mix, street connectivity; presence of high-quality parks | Overweight based on CDC growth charts | Age, sex, race, parent age, parent education, parent BMI, household income; neighbourhood characteristics | Environments more conducive to walking associated with obesity | 9 |
| Sallis, 2018^50^ | cross-sectional | USA | 928 | 12-16 | Walkability index: residential density, street connectivity, retail floor area ratio, land use mix | BMI percentile and weight status based on CDC growth charts (height and weight self-reported) | Age, sex, race/ethnicity, days per at primary address, schooling status, work status, driver's license | No statistically significant association | 8 |
| Shahid, 2015^37^ | cross-sectional | Canada | 37,460 | 4-6 | Walk Score: straight line distance to local amenities (education, recreational, food, retail, and entertainment). Distances are summed and weighted by a distance decay function | BMI | NA | Associated of walkability with childhood obesity driven by low socio- economic status, lower education attainments, and recent immigration | 9 |
| Slater, 2013^46^ | cross-sectional | USA | 11,041 | 13, 15, 17 | Walkability index: streets with mixed land use, sidewalks, sidewalk buffers, sidewalk/street lighting, other sidewalk elements (e.g., sidewalk continuity, shade), traffıc lights, pedestrian signal at traffic light, marked crosswalks, pedestrian crossing and other signage, and public transit. | BMI, weight status based on CDC growth charts (height and weight self-reported) | Age, sex, race/ethnicity, grade, parental education, community physical disorder scale, presence of bike lanes, presence of off-road trails, student perception of safety going to and from school, community-level median household income, community compactness index | More-walkable communities associated with reduced prevalence of adolescent overweight and obesity | 10 |
| Spence, 2008^38^ | cross-sectional | Canada | 501 | 4-6 | Walkability index: intersection density, dwelling density, land use mix; intersection density weighted twice | BMI, weight status based on CDC growth charts and IOTF cut-offs | Age, sex, neighbourhood-level education, proportion of employed women, physical activity, junk food consumption | Negative associations of walkability and intersection density with weight status in girls, not significant in boys. | 9 |
| Stowe, 2019^45^ | cross-sectional | USA | 13,469 | 7–14 | Walk Score: intersection density, block length, distance to diverse amenities | BMI *z*-scores | Age, sex, race/ethnicity, urbanicity | Walk Score positively associated with BMI *z*-score among urban youth and negatively associated with BMI *z*-score among rural youth; no association urban–rural mixed areas | 11 |
| Wilding, 2019^23^ | longit. | UK | 14,084 and 5,637 at the two follow-ups | 4,5, 10,11 | Walkability index: residential density, gradient change, intersection density, land-use mix; intersection density weighted twice | BMI centiles based on age-sex specific 1990 UK weight reference | Maternal BMI, age, education, ethnicity, smoking at the  start of pregnancy and parity, spatial clustering | Walkability positively associated with the risk of being overweight or obese at ages 10–11 | 11 |
| Xue, 2020^43^ | longit. | USA | 44,100 and 43,800 at follow ups | 11-17 | Population-weighted intersection density at the census tract level at baseline and follow-up | BMI, weight status based on CDC growth charts (height and weight parent-reported) | Area-level poverty rate percentage of urban areas, population density, racial/ethnic composition measured by Shannon entropy index | Associations of intersection density and childhood obesity prevalence | 10 |
| Yang, 2018^52^ | cross-sectional | USA | 41,283 | 3-18 | Walk score, street intersection density, population density | Weight status based on CDC growth charts | Age, sex, race, economically disadvantaged status, school type, and school level | Negative association of population density with the risk of overweight and obesity, but not intersection density or Walk Score | 11 |

longit: longitudinal study design, QA: quality assessment. References relate to list of references in the main manuscript.

**Table S4d. Characteristics of studies included in the systematic review on accessibility and availability of parks and playgrounds**

| First Author, Year | Study design | Country | Sample size | Age (years) | Exposure | Outcome | Confounders | Main findings | QA |
| --- | --- | --- | --- | --- | --- | --- | --- | --- | --- |
| Armstrong, 2015^53^ | longit. | USA | 93 | 8-14 | Park density, 10 miles circular buffers from home addresses | BMI *z*-score | Age, sex, treatment condition | Association of increased park density with decreased BMI *z*-score over time behavioral intervention group, not in the control group. | 8 |
| Bloemsma, 2019^18^ | longit. | Netherlands | 3,680 | 3-17 | NDVI, 300 m and 3,000 m circular buffers from home address; percentages of green space; distance to the nearest park; at time of measurements | BMI, weight status based on IOTF cut-offs (height and weight parent-reported) | Age, sex, maternal and paternal level of education, maternal smoking during pregnancy, parental smoking in the child's, home and neighborhood socioeconomic status, region | Association of NDVI and total percentage of green space (3000 m buffer) with weight status after adjustment for age and sex, distance to the nearest park with lower odds of being overweight in urban areas | 10 |
| Dadvand, 2014^63^ | cross-sectional | Spain | 3,178 | 9-12 | NDVI, 100 m, 250 m, 500 m, and 1,000 m circular buffer from home address; within 300 m of a forest or a park | BMI z-scores, weight status based on age-sex specific WHO growth chart (height and weight self-reported) | Highest educational achievement by either parent, the type of school, area-level socioeconomic status using quintiles of the Urban Vulnerability index | Significant association of higher greenness with lower prevalence of overweight/obesity (all buffer sizes) and with lower BMI z-scores (100 m buffer) | 9 |
| Goldsby, 2016^58^ | longit. (quasi-experimental) | USA | 1,443 | 2-17 | Euclidian distance to a newly built park from the home address | BMI z*-*score, weight status based on age-sex specific CDC growth chart | Age, gender, race, ethnicity, payer type | No association | 10 |
| Gutierrez-Zornoza, 2015^64^ | cross-sectional | Spain | 956 | 10-12 | Distances from home to facilities (green spaces and sports facilities) measured along the road network | BMI percentage fat mass, waist circumference; weight status based on IOTF cut-offs | Age, commuting, cardiovascular fitness | No significative associations | 9 |
| Hsieh, 2015^47^ | cross-sectional | USA | 576 | 8-18 | Park access defined as aggregate park space in acres within walking-distance buffers from home address | Percentage body fat, waist circumference, BMI *z*-score based on age-sex specific CDC growth chart | Age, Tanner stage, neighborhood socio-cultural characteristics, spatial autocorrelation | Association of increased park access with lower percentage body fat in girls | 8 |
| Morgan Hughey, 2017^69^ | cross-sectional | USA | 13,469 | 8-11 | Neighbourhood park and playground availability based on number of each facility within or intersecting each youth's Census block group | BMI percentile | Age, sex, socioeconomic status, race/ethnicity, block group characteristics including population, racial/ethnicity minority, median household income | Significant association of number of parks with lower BMI in girls, but not in boys | 11 |
| Lange, 2011^61^ | cross-sectional | Germany | 3,440 | 13-15 | Number of parks and sport fields within districts in Kiel | BMI, weight status based on age-sex specific and German reference curves | Age, sex, socioeconomic status | No association | 10 |
| Lovasi, 2011^48^ | cross-sectional | USA | 428 | 2–5 | Street tree density, park access, playground access, 500 m circular buffers from home addresses and Head Start centre (children living < 2 km from Head Start centre) or 500m buffer straight line between the child’s home and Head Start centre (children living > 2 km centre) | skinfold thicknesses; BMI *z*-score; weight status based on age-sex specific CDC growth chart | Age, sex, race/ethnicity, mother’s age, birth outside of the USA, use of Spanish, and employment status, number of rooms in the home, neighborhood characteristics; total number of hours recorded as awake and for season | Significant association of park access with sum of skinfold | 8 |
| Lovasi, 2013^70^ | cross-sectional | USA | 11,562 | 3-5 | Density of street trees and park area within 400 m circular buffer from ZIP code | BMI *z*-score based on age-sex specific CDC growth chart | Age, sex, race, ethnicity, neighborhood characteristics | Association of higher street tree density lower prevalence of obesity, no other associations | 10 |
| Manandhar, 2018^67^ | cross-sectional | Nepal | 440 | 6-13 | Distance between home address to nearest open space | Overweight and obesity based on age-sex specific WHO growth reference | Sex, age, wealth status, type of family, parity, mother’s occupation and education, consumption of sweetened beverage and snack, television habits, mode of travel to school, level of physical activity | Significant association of distance to green space with childhood overweight and obesity. Children whose residence was >1 km away from green space at higher risk of overweight/obesity | 8 |
| McCarthy, 2017^71^ | cross-sectional | USA | 13,469 | 8-11 | Playgrounds within a 0.5 mile network buffer | BMI percentiles based on age-sex specific CDC growth chart | Age, sex, race/ethnicity, socioeconomic status, total population of block group | No association | 11 |
| Nesbit, 2014^73^ | cross-sectional | USA | 39,542 | 11-17 | Presence of parks and playgrounds | Weight status based on age-sex specific CDC growth chart (height and weight parent-reported) | Television habits, computer time, parent report of safety and trust that people will help the child | Significant association of parks and playgrounds with weight status | 9 |
| Ohri-Vachaspati, 2013^68^ | cross-sectional | USA | 702 | 3-18 | Presence or absence of parks in 0.25, 0.5, 1 miles buffers; distance to the nearest park | Weight status based on age-sex specific CDC growth chart (height and weight parent-reported) | Age, sex, race/ethnicity, mother’s educational level, primary language spoken at home, nativity, household poverty status, parent’s BMI, neighborhood socioeconomic status. | Significant associations of presence of a large park with weight status, no association for distance to the nearest park | 8 |
| Petraviciene, 2018^65^ | cross-sectional | Lithuania | 1,489 | 4-6 | NDVI at home address; distance to a nearest city park | Overweight/obesity based on international body mass index cut-off points | Sex, birth weight, sedentary behavior, family status, maternal age, education, employment status, smoking during pregnancy, secondhand smoking, mother-child relations, NO_2_ | Association of higher NDVI level with reduced risk of being overweight/obese, effect modification by socioeconomic status and distance to parks | 9 |
| Poole, 2017^59^ | cross-sectional | UK | 2,712 | 4-5 | Number of purposefully constructed play areas within 1 km (density); distance to nearest play area from residential postcode | Weight status | Sex, play area quality, neighbourhood socioeconomic status | No association | 10 |
| Potestio, 2009^74^ | cross-sectional | Canada | 6,772 | 3-8 | Number of parks/green spaces per 10,000 residents; percentage of parks/green space area within a community; average distance to park/green space; the proportion of parks/green space service area as a proportion of the total area within a community | BMI, overweight or obese status based Cole’s international age-sex specific cut-offs | Sex, dissemination area, median family income, community-level education and proportion of visible minorities | No association | 10 |
| Potwarka, 2008^66^ | cross-sectional | Canada | 108 | 2-17 | Number of parks (based on park centroid) within 1 km of home, total area of parkland within 1 km, and distance to the closest park from home. | Weight status based on age-sex specific CDC growth chart (height and weight parent-reported) | Sex, age, parent’s BMI | Children with a park playground within 1 km were ~ five times more likely to be healthy weight, no association with proximity-based park metric | 8 |
| Sanders, 2015^54^ | longit. | Australia | 4,423 | 6-13 | Proportion of neighbourhood green space | Waist circumference and waist-to-height ratio | Socioeconomic status, stratified by sex, effect modification by age was explored | Significative association of green space with changes in waist circumference and changes in waist-to-height ratio in boys. | 10 |
| Sanders, 2015^55^ | longit. | Australia | 4,423 | 6-13 | Objective measure of green space availability | BMI every 2 years | Family income, Australian Indigenous status, mothers’ education, language spoken | Association of BMI with green space modified by age and gender: older boys with little to no green space have higher BMI trajectories than those living in areas with modest or high amounts of green space with little additional benefit beyond a modest quantity of green space. Associations for girls, largely attenuated after adjusting for socio-economic confounders. | 10 |
| Schalkwijk, 2017^60^ | cross-sectional | UK | 6,467 | 7 | Neighbourhood ranking based on green space deciles | Overweight/obesity at age seven based on Cole’s international age-sex specific cut-offs | Food consumption, physical activity, rules and regularity, socioeconomic status explored as moderators or mediators | Statistically significant associations of low levels of green space, no access to garden, run down area and childhood overweight/obesity | 10 |
| Schule, 2016^62^ | cross-sectional | Germany | 3,499 | 5-7 | Playground and park space for infants and children within each school district. | Age-sex-specific BMI percentile and overweight /obese status based on IOTF cut-offs | Sex, birth weight, maternal BMI, paternal BMI, paternal education, paternal working status, income, crowding, neighbourhood socioeconomic status | Age-specific playground space, and public park availability not independently associated with overweight | 10 |
| Van der Zwaard, 2018^56^ | longit. | UK | 6,001 | 3-11 | Neighbourhood ranking based on green space and garden deciles | BMI, overweight status | Age, sex, education level of the main carer | Statistically significant associations of more gardens with lower BMI, no effects on odds of overweight | 10 |
| Wall , 2012^75^ | cross-sectional | USA | 2,682 | 12-16 | Percentage area of park/recreation space | BMI *z*-scores and obesity condition | Age, socioeconomic status, race/ethnicity | Decrease in park/recreation space associated with higher BMI z*-*score in both boys and girls. | 10 |
| Wasserman, 2014^72^ | cross-sectional | USA | 12,118 | 4-12 | Density of parks based on counts within 1 mile buffer from school | BMI percentiles | Age, sex, public school, population, White ethnicity, population change | Inverse association of number of parks and fitness centers with BMI percentile | 11 |
| Wilding, 2019^29^ | longit. | UK | 14,084 and 5,637 at the two follow-ups | 4-5 and 10-11 years | Local access to natural greenspaces at the time of birth | BMI centiles based on age-sex specific 1990 UK weight reference | Age, education, ethnicity, maternal BMI, smoking at the start of pregnancy, parity | Inverse association of access to natural green spaces with becoming overweight or obese by age 10–11 | 11 |
| Wolch, 2011^57^ | longit. | USA | 3,173 | 9-18 | Park space (in acres), 500 m circular buffer from home address | BMI; attained BMI growth at age 18 | Traffic density (150m buffer), average urban imperviousness, NDVI, road lengths, intersection density (500m buffer), population living below poverty live | Park space significantly inverse association with attained BMI at age 18, effect sizes larger for boys than for girls | 10 |
| Yang, 2018^52^ | cross-sectional | USA | 41,283 | 3-18 | Population-weighted distance to the nearest park | Overweight and obesity based on CDC growth charts | Age, sex, race, economically disadvantaged status, school type, school level | Positive association of distance to parks and boys' risk of being with overweight or obesity | 11 |

longit: longitudinal study design, QA: quality assessment. References relate to list of references in the main manuscript.

**Table S5. Quality assessment scores using modified Newcastle-Ottawa Scale**

| **Noise** | Author, year | S1 | S2 | S3 | S4 | C1 | O1 | O2 | Score |
| --- | --- | --- | --- | --- | --- | --- | --- | --- | --- |
|  | Bloemsma, 2019^18^ | * | * | ** | * | ** | * | * | 9 |
|  | Christensen, 2016^19^ | * | * | ** | ** | ** | * | * | 10 |
|  | Wallas, 2019^20^ | * | * | ** | * | ** | ** | * | 10 |
|  | Weyde, 2018^21^ | * | * | ** | * | ** | * | * | 9 |
| **Air pollution** | Alderete, 2017^24^ | * | * | ** |  | ** | ** | * | 9 |
|  | Bloemsma, 2019^18^ | * | * | ** | * | ** | * | * | 9 |
|  | Chiu, 2017^31^ | * | * | ** |  | ** | ** | * | 9 |
|  | deBont, 2019^26^ | * | * | ** | * | ** | ** | * | 10 |
|  | Dong, 2014^22^ | * | * | ** | ** | ** | ** | * | 11 |
|  | Fioravanti, 2018^33^ | * | * | ** |  | ** | ** | * | 9 |
|  | Fleisch, 2019^32^ | * | * | ** | * | ** | ** | * | 10 |
|  | Frondelius, 2018^28^ | * | * | ** | * | ** | ** | * | 10 |
|  | Huang, 2019^34^ | * | * | ** | * | ** | ** | * | 10 |
|  | Jerret, 2014^27^ | * | * | ** | * | ** | ** | * | 10 |
|  | Kim, 2018^30^ |  | * | ** | * | ** | ** | * | 9 |
|  | Mao, 2017^35^ | * | * | ** | * | ** | ** | * | 10 |
|  | McConnell, 2015^25^ | * | * | ** | * | ** | ** | * | 10 |
|  | Wilding, 2019^23^ | * | * | ** | ** | ** | ** | * | 11 |
| **Neighbourhood walkability** | Colley, 2019^39^ | * | * | ** | ** | ** | ** | * | 11 |
|  | Duncan, 2014^36^ | * | * | ** | ** | ** | ** | * | 11 |
|  | Gose, 2013^40^ | * | * | ** |  | ** | ** | * | 9 |
|  | Hagani, 2019^42^ | * | * | ** |  | ** | ** | * | 9 |
|  | Hsieh, 2015^47^ |  | * | ** |  | ** | ** | * | 8 |
|  | Jia, 2019^51^ | * | * | ** | * | ** | ** | * | 10 |
|  | Kligerman, 2007^49^ | * | * | ** |  | ** | ** | * | 9 |
|  | Lovasi, 2011^48^ |  | * | ** |  | ** | ** | * | 8 |
|  | Lovasi, 2013^70^ |  | * | ** | ** | ** | ** | * | 10 |
|  | Molina-Garcia, 2017^41^ | * | * | ** |  | ** | ** | * | 9 |
|  | Saelens, 2012^84^ | * | * | ** |  | ** | ** | * | 9 |
|  | Sallis, 2018^50^ | * | * | ** |  | ** | * | * | 8 |
|  | Shahid, 2015^37^ | * | * | ** | ** |  | ** | * | 9 |
|  | Slater, 2013^46^ | * | * | ** | ** | ** | * | * | 10 |
|  | Spence, 2008^38^ | * | * | ** |  | ** | ** | * | 9 |
|  | Stowe, 2019^45^ | * | * | ** | ** | ** | ** | * | 11 |
|  | Wilding, 2019^23^ | * | * | ** | ** | ** | ** | * | 11 |
|  | Xue, 2020^43^ | * | * | ** | ** | ** | * | * | 10 |
|  | Yang, 2018^52^ | * | * | ** | ** | ** | ** | * | 11 |
| **Availability and accessibility of parks and playgrounds** | Armstrong, 2015^53^ |  | * | ** |  | ** | ** | * | 8 |
|  | Bloemsma, 2019^18^ | * | * | ** | * | ** | ** | * | 10 |
|  | Dadvand, 2014^63^ | * | * | ** | * | ** | * | * | 9 |
|  | Goldsby, 2016^58^ | * | * | ** | * | ** | ** | * | 10 |
|  | Gutierrez-Zornoza, 2015^64^ | * | * | ** |  | ** | ** | * | 9 |
|  | Hsieh, 2015^47^ |  | * | ** |  | ** | ** | * | 8 |
|  | Morgan Hughey, 2017^69^ | * | * | ** | ** | ** | ** | * | 11 |
|  | Lange, 2011^61^ | * | * | ** | * | ** | ** | * | 10 |
|  | Lovasi, 2011^48^ |  | * | ** |  | ** | ** | * | 8 |
|  | Lovasi, 2013^70^ |  | * | ** | ** | ** | ** | * | 10 |
|  | Manandhar, 2018^67^ | * | * | * |  | ** | ** | * | 8 |
|  | McCarthy, 2017^71^ | * | * | ** | ** | ** | ** | * | 11 |
|  | Nesbit, 2014^73^ | * | * | * | ** | ** | * | * | 9 |
|  | Ohri-Vachaspati, 2013^68^ | * | * | ** |  | ** | * | * | 8 |
|  | Petraviciene, 2018^65^ | * | * | ** | * | ** | * | * | 9 |
|  | Poole, 2017^59^ | * | * | ** | * | ** | ** | * | 10 |
|  | Potestio, 2009^74^ | * | * | ** | * | ** | ** | * | 10 |
|  | Potwarka, 2008^66^ | * | * | ** |  | ** | * | * | 8 |
|  | Sanders, 2015^56^ | * | * | ** | * | ** | ** | * | 10 |
|  | Sanders, 2015^53^ | * | * | ** | * | ** | ** | * | 10 |
|  | Schalkwijk, 2017^60^ | * | * | ** | * | ** | ** | * | 10 |
|  | Schule, 2016^62^ | * | * | ** | * | ** | ** | * | 10 |
|  | Van der Zwaard, 2018^56^ | * | * | ** | * | ** | ** | * | 10 |
|  | Wall, 2012^75^ | * | * | ** | * | ** | ** | * | 10 |
|  | Wasserman, 2014^72^ | * | * | ** | ** | ** | ** | * | 11 |
|  | Wilding, 2019^29^ | * | * | ** | ** | ** | ** | * | 11 |
|  | Wolch, 2011^57^ | * | * | ** | * | ** | ** | * | 10 |
|  | Yang, 2018^52^ | * | * | ** | ** | ** | ** | * | 11 |

References relate to list of references in the main manuscript.


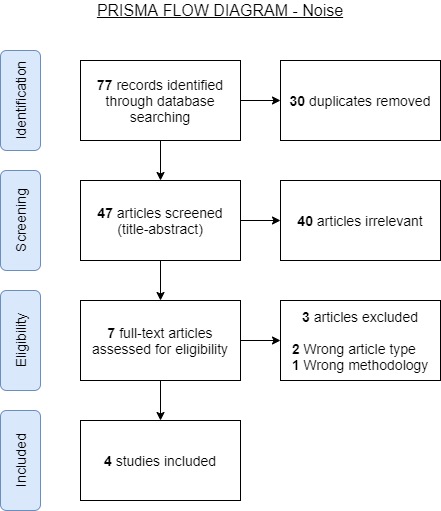


**Figure S1. PRISMA flow diagram for traffic noise and childhood obesity**


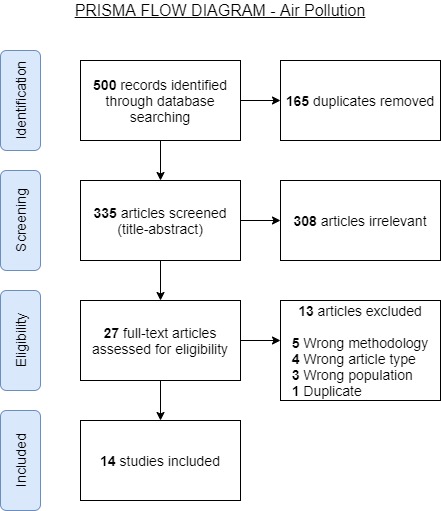


**Figure S2. PRISMA flow diagram for air pollution and childhood obesity**


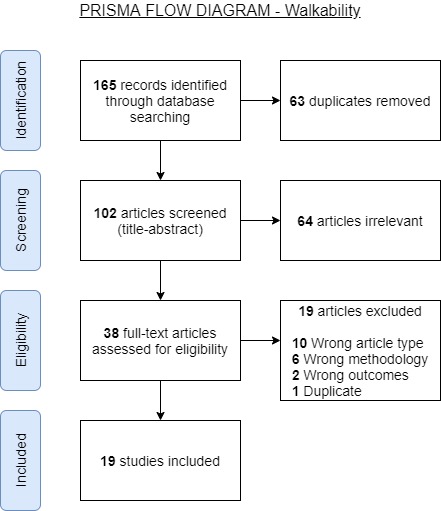


**Figure S3. PRISMA flow diagram for neighbourhood walkability and childhood obesity**


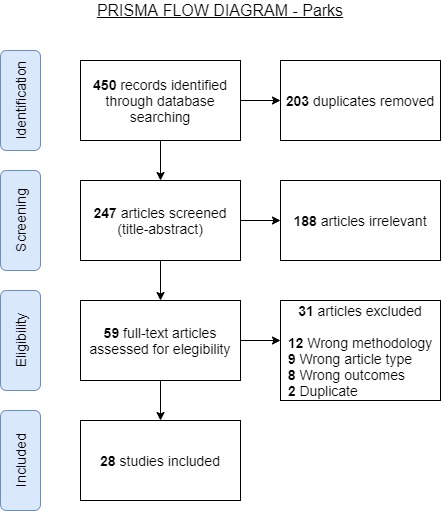


**Figure S4. PRISMA flow diagram for availability and accessibility of parks and playgrounds and childhood obesity**


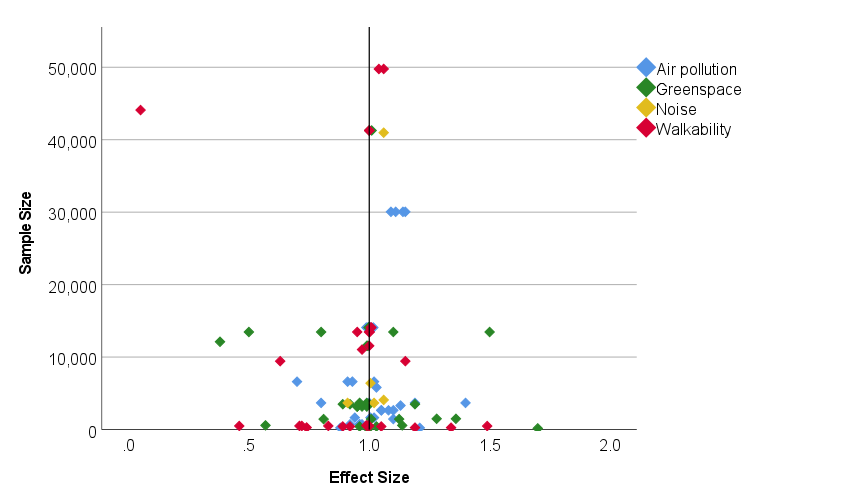


**Figure S5. Publication bias by built environment characteristics**
